# Slow Cortical Potential Neurofeedback in Chronic Tinnitus Therapy: A Case Report

**DOI:** 10.1007/s10484-015-9318-5

**Published:** 2015-10-12

**Authors:** Rafał Milner, Monika Lewandowska, Małgorzata Ganc, Katarzyna Cieśla, Iwona Niedziałek, Henryk Skarżyński

**Affiliations:** Department of Experimental Audiology, World Hearing Center, Institute of Physiology and Pathology of Hearing, Mokra 17 Str., Kajetany, 05-830 Nadarzyn, Poland; Bioimaging Research Center, World Hearing Center, Institute of Physiology and Pathology of Hearing, Mokra 17 Str., Kajetany, 05-830 Nadarzyn, Poland; Department of Audiology and Phoniatrics, World Hearing Center, Institute of Physiology and Pathology of Hearing, Mokra 17 Str., Kajetany, 05-830 Nadarzyn, Poland; World Hearing Center, Institute of Physiology and Pathology of Hearing, Mokra 17 Str., Kajetany, 05-830 Nadarzyn, Poland

**Keywords:** Tinnitus therapy, Slow cortical potential Neurofeedback, Quantitative electroencephalography

## Abstract

This study is the first to demonstrate outcomes of slow cortical potential (SCP) Neurofeedback training in chronic tinnitus. A 50-year old male patient with tinnitus participated in three SCP training blocks, separated with 1-month breaks. After the training the patient reported decreased tinnitus loudness and pitch, as well as improved quality of daily life. A quantitative electroencephalography analysis revealed close to normal changes of resting state bioelectrical activity in cortical areas considered to be involved in tinnitus generation. The present case study indicates that SCP Neurofeedback training can be considered a promising method for tinnitus treatment.

## Introduction

Tinnitus is a phantom auditory percept. The patient hears an internal sound in the absence of an external sound source. Tinnitus often co-occurs with a hearing impairment, hyperacusis, problems with concentration, nervous strain, sleep disorders, headache, excessive stress, anxiety disorders and depression (Crönlein et al. 2011; Landgrebe and Langguth 2011a; Langguth et al. 2011).

The prevalence of chronic tinnitus in the global population is estimated at 10–15 %, and about 6–25 % of patients experience severe tinnitus affecting their lifestyle and significantly reducing their quality of life (Axelsson and Ringdahl 1989; Baguley 2002; Eggermont and Roberts 2004; Heller 2003). Data collected at the Institute of Physiology and Pathology of Hearing (Warsaw, Poland) (IPPH) indicate that these numbers are similar in Poland, with about 5 % of the Polish population reporting persistent tinnitus (Bartnik 2010).

Despite this, there is no single generally approved concept elucidating the mechanisms of tinnitus. Historically, it had been thought as originating from the ear itself (see review in: Møller 2011). However, in 1987 Jürgen Tonndorf pointed to similarities between tinnitus and phantom pain perception and proposed a neurophysiological model of tinnitus generation in the central nervous system (CNS). Since then many new theories have been developed to explain the aetiology of tinnitus (De Ridder 2011; Schlee et al. 2011). Most have converged with the Tonndorf model and showed that tinnitus probably occurs due to an abnormal neuronal activity caused by auditory deprivation (Jastreboff 1990, 1995). The concept that has gained the most attention implies a thalamocortical dysrhythmia in tinnitus (De Ridder et al. 2011, 2013; Llinás et al. 1999). According to this model tinnitus is caused by an abnormal, spontaneous gamma band activity (>30 Hz) generated in the auditory cortex as a consequence of hyperpolarization of the thalamic nuclei resulting from auditory deafferentation. In normal conditions sounds increase thalamocortical rhythms to gamma band activity (Joliot et al. 1994). In contrast, in tinnitus (in the deafferented state) the oscillatory activity in the thalamocortical circuits decreases from resting state alpha activity (8–12 Hz) to theta band activity (4–7 Hz) (De Ridder 2007; Steriade 2006). As a consequence lateral inhibition is reduced, inducing gamma band activity in brain regions adjacent to those deprived of auditory input. This phenomenon is known as the ‘edge effect’ (De Ridder et al. 2011, 2013; Llinás et al. 1999, 2005).

The complex nature and the yet unexplored aetiology of tinnitus make the diagnostic assessment difficult. Although clinical practice offers a number of various treatment methods for tinnitus, no one is entirely effective. If the cause of tinnitus is easily recognizable, e.g. the condition is triggered by an organic damage or a dysfunction in the middle ear, inner ear or neural pathways transmitting acoustic information from the ear to the central auditory structures, a specific medical intervention, such as pharmacological or surgical can be applied. In most instances, however, tinnitus is idiopathic and only non-pharmacological treatment methods can be recommended (e.g. De Ridder 2007; Duckro et al. 1984; Flor et al. 2004; Langguth and De Ridder 2013).

One such therapy is the Neurofeedback technique which has already been proven effective in the treatment of tinnitus (Crocetti et al. 2011; Dohrmann et al. 2007; Hartmann et al. 2013). An example study by Dohrmann et al. (2007) demonstrated that the delta/alpha ratio Neurofeedback protocol could bring about a significant improvement in subjective perception of tinnitus. It has been shown that the power of delta and alpha frequency bands is abnormally changed in patients with tinnitus (with alpha decreased and delta increased), as measured in the right temporal lobe and the left frontal lobe. A correlation between the power of alpha and delta, the frequency index of both bands and the tinnitus-related distress (evaluated with the Tinnitus Questionnaire) has also been found in tinnitus patients (Weisz et al. 2005). A simultaneous delta-down and alpha up-training could therefore significantly reduce loudness of tinnitus and normalize brain activity underlying tinnitus generation (Dohrmann et al. 2007).

A special type of the Neurofeedback training, using slow cortical potentials (SCP Neurofeedback), has been successfully employed in the treatment of different brain dysfunctions, such as epilepsy, ADHD and migraines (Kotchoubey et al. 2001; Siniatchkin et al. 2000a; Strehl et al. 2006b). Slow cortical potentials are direct-current shifts of the EEG signal, generated in the upper cortical layer. SCPs are considered event-related potentials, as they are not oscillatory in nature but induced by external or internal events. They last from 0.3 s up to several seconds and their polarity can be either positive or negative (Strehl et al. 2006b). A series of experiments performed in the 80s and 90s by a scientific team led by Niels Birbaumer and Thomas Elbert indicate that SCPs are related to cortical responsiveness (Birbaumer 1999; Birbaumer et al. 1990). Accordingly, negative SCPs are associated with the depolarization of large cortical cell assemblies and the reduction of their excitation thresholds. Their occurrence is believed to be a manifestation of an “activation state” and preparation of particular brain regions to perform a task. Positive SCPs are believed to correspond to decreased cortical excitability and an increased excitation threshold of neuronal populations. Therefore, they are considered to be related to inhibitory processes in cortical networks (Elbert 1986; Kotchoubey 2000; Lutzenberger et al. 1979; Rockstroh and Elbert 1990; Strehl et al. 2006a, b).

According to the authors’ best knowledge, there have not been any studies published to date applying SCP Neurofeedback training in tinnitus patients. This paper presents a single case study of a patient who participated in a series of specifically designed training sessions during which he practiced the self-regulation of SCPs. As previously described, tinnitus can be related to abnormal neuronal synchronization, typically at high frequency ranges, in various cortical regions (see e.g. De Ridder et al. 2011; Eggermont 2003; Van Der Loo et al. 2009). Furthermore, some studies suggest a significantly decreased excitation threshold of cortical neurons in patients, as compared to healthy individuals (Kropp et al. 2012; Shiraishi et al. 1991). In the current work we hypothesize that acquiring the ability to regulate cortical excitability would elevate the abnormally low neuronal excitation thresholds and restore cortical excitation-inhibition homeostasis. This normalization should, in turn, attenuate the undesired neuronal synchronization underlying tinnitus and decrease or completely cancel out the tinnitus sensation. Therefore, the aim of the present study was to verify whether SCP Neurofeedback training protocol could be used to suppress tinnitus sound and alleviate tinnitus-related symptoms.

## Case Report

Patient, Z.C., a 50-year-old male, provided written informed consent for the publication of this case study. He had suffered from chronic tinnitus since 2006 and described it as a high-intensity and a relatively loud sound occurring in both ears and/or inside the head. At first tinnitus was transient but over time it turned into a chronic sensation of a high-frequency squeak. Z.C. described the condition as extremely bothersome, impeding falling asleep, and becoming even more intensive under stress.

The severity of the experienced symptoms motivated Z.C. to visit the Institute of Physiology and Pathology of Hearing in Warsaw (IPPH) in 2007. Audiological assessment [pure tone audiometry, impedance audiometry, auditory brainstem responses (ABR)], together with MR and CT exams revealed normal hearing and lack of any organic aetiology of tinnitus. During the next 3 years, Z.C. tested various methods to alleviate the symptoms, including the tinnitus retraining therapy (TRT) (Jastreboff 2007), but none of them was effective. Moreover, Z.C. claimed that tinnitus increasingly complicated his life, by making him more nervous, hindering work, weakening concentration, and resulting in a large number of mistakes. As a consequence the patient lost his job.

To counteract the symptoms, Z.C. was referred to a psychiatric consultation, followed by prescription of sedative and SSRI medication. However, the patient did not accept this form of therapy, as he was afraid of the possible side effects. Finally, SCP Neurofeedback training was offered which the patient started at IPPH in 2013.

### Clinical Assessment Before SCP Neurofeedback Training

Prior to SCP Neurofeedback training Z.C. was in a relatively good health and was not using any medication at least 3 months before and throughout the whole duration of the training. The following diagnostic tests: (a) standard and high-frequency pure tone audiometry (0.125–16 kHz), (b) Tinnitus Handicap Inventory (THI) (Newman et al. 1996) and Beck Depression Inventory (BDI) (Beck et al. 1961) were administered to the patient. The structure of the tests and the patient’s outcomes have been described below.

#### Standard and High-Frequency Pure Tone Audiometry

A standard and a high-frequency pure tone audiometry (PTA and HFA, respectively) were performed by an expert audiologist in a sound-proof chamber according to ISO 8253-1 (1989) standards and recommendations by the British Society of Audiology (2011). All measurements were performed with the use of a Madsen Astera audiometer (GN Otometrics, Germany) and Sennheiser HDA-200 headphones (Sennheiser Electronic GmbH & Co. KG, Germany) (ISO 389-8 2004; ISO 389-5 2006; IEC 60645-1 2012). The standard PTA thresholds were determined for octave frequencies of 0.25, 0.5, 1, 2, 4, 6, and 8 kHz. HFA thresholds included frequencies of 9, 10, 11.2, 12.5 and 16 kHz. Both tests were performed according to a modified ascending Hughson–Westlake procedure (Carhart and Jerger 1959). The test started with a presentation of a tone at one of the tested frequencies and at a clearly audible (but not loud) sound level (e.g. 40 dB SPL). The task of the patient was to press a special button when a tone was heard. If the patient was able to detect the tone, the sound level was reduced by 10 dB; if not, the sound level was increased by 5 dB. This procedure was repeated several times, until a 50 % response rate was obtained for a given level of the test signal and until hearing thresholds for all tested frequencies were determined.

The results of the standard PTA in the patient revealed normal pure tone hearing thresholds in both ears lower than or equal to 20 dB HL at 0.5–4 kHz and ≤40 dB HL at 8 kHz (WHO 2008). The HFA measurements demonstrated age normal hearing thresholds for all tested high frequencies in both ears, lower or equal to 50 dB HL (Rodriquez Valiente et al. 2014).

#### Tinnitus Handicap Inventory

An experimental Polish version of the Tinnitus Handicap Inventory was completed by the patient before the training. This test is used for self-evaluation of the influence of tinnitus on three tiers of daily functioning, involving functional, emotional and catastrophic reactions. There are 25 items in THI. On a 3-point scale (Yes—4 points, Sometimes—2 points, No—0 points), the respondent assesses the extent to which an item applies to his/her experience of tinnitus. Five grades of tinnitus handicap are distinguished by calculating the total score: Grade 1 (0–16 points)—slight or no handicap, Grade 2 (18–36 points)—mild handicap, Grade 3 (38–56 points)—moderate handicap, Grade 4 (58–76 points)—severe handicap, Grade 5 (78–100 points)—catastrophic handicap. The original version of the test has excellent internal consistency reliability (Cronbach’s alpha = 0.93), as well as an adequate convergent (Tinnitus Handicap Questionnaire) and discriminant (Beck Depression Inventory) validity (Newman et al. 1996). The test–retest reliability of THI is good (correlation r >0.8, between test and retest administrations; 95 % score differences within ±2 SD (Newman et al. 1998). Patient Z.C. obtained 40 out of 100 maximum points in the THI, which would suggest moderate levels of handicap.

#### Beck Depression Inventory (BDI)

An experimental Polish version of BDI I was used for self-evaluation of occurrence and severity of depression symptoms (Beck et al. 1961, Porowski and Jernajczyk 1977). The Beck Depression Inventory has 21 items which describe feelings of pessimism, guilt, failure, irritation, insomnia, weight loss, etc. Each item is addressed on a 0–3 point scale. The overall score of ≤19 points is considered to indicate no or mild, 20–28 points—moderate, and ≥29—severe depressive symptoms. The original BDI I has high internal consistency reliability (Cronbach’s alpha = 0.80) and test–retest reliability (0.93 correlation, *p* < 0.001). The mean correlations of BDI and the Hamilton Psychiatric Rating Scale for Depression (convergent validity) are in the range 0.60–0.74 (Beck et al. 1988). The patient had a score of 9/63 points in the BDI I, what indicates lack of depressive symptoms.

### SCP Neurofeedback Training

SCP Neurofeedback training was designed in compliance with standards available in literature (e.g. Strehl 2009) and performed by means of Biograph Infinity 5.0. software (Thought Technology Ltd). The procedure comprised three 2-week training blocks. Each block involved 10 × 1.5 h sessions from Monday to Friday. A single session consisted of 4 runs, each comprising 40 trials. There were 3 feedback and 1 transfer (with no feedback provided) runs performed in each session (see next paragraphs for a more detailed description). The whole training period was approximately 3.5 months, with breaks of around 1 month in length between blocks, during which the patient was encouraged to train at home without using the Neurofeedback equipment (details are provided in paragraph *Transfer of self*-*regulation training into everyday life*).

#### Feedback Runs

**Fig. 1:**
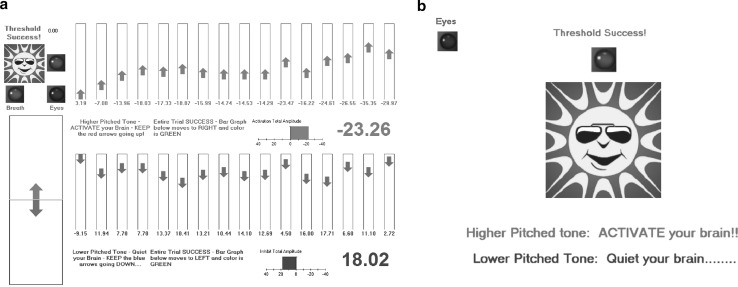
Visual Displays presented to the patient during SCP Neurofeedback training **a** Feedback runs. *Red arrows* indicate momentary SCP amplitude values (registered every 0.5 s) generated during negative feedback trials; *blue arrows* indicate momentary SCP amplitude values generated during positive feedback trials. Values below the *arrows* on the *right side* represent the mean amplitude of SCPs generated in a single trial. *Arrows* in the *bottom left* *corner* moved during each trial and indicated the required SCP shift direction. An audio-visual stimulus: a *rotating sun animation*, an *applause sound* and a *green light* turning on in the *upper left corner*, served as a reward. The reward was presented to the patient after completing a successful trial when the adequate threshold of the mean absolute amplitude of SCPs was exceeded (for a detailed description of the set success threshold see section “Feedback Runs”). **b** Transfer runs. Momentary and mean amplitudes of SCPs generated in transfer trials were not presented to the patient. The only reward for the patient was the audio-visual stimulus: a *rotating sun animation* moving in the middle of the screen accompanied with an *applause* and a *green control light* turning on above it. For both types of trials, a *red control light* in the *top left corner* of the training Visual Display, linked to the EOG channel, was used by the patient to monitor his eye movements. The *red control light* turned on when the movement was too intensive (Color figure online)

During feedback runs each single trial was performed for 8 s. Red and blue arrows (pointing upwards and downwards, respectively) were shown on the training Visual Display (Fig. 1a). Z.C. was asked to keep the track of the arrows. He was also presented with either two high- or two low-frequency tones. The first and second tone marked the beginning and the end of a trial, respectively. If a low-frequency tone occurred, the patient was required to move consecutive red arrows upwards by producing cortical negativity (negative SCP trials) (see Fig. 1a). The arrows appeared every 0.5 s showing momentary amplitude values of the generated SCPs. Analogically, after hearing a high-frequency tone Z.C. was asked to move blue arrows downwards which corresponded with suppressing negativity or increasing positivity (positive SCP trials). The amplitude of the SCP was calculated with reference to a 2 s pre-trial period. Z.C. was kept informed on his progress in the task. In addition, the mean SCP amplitude value achieved during the whole trial was provided. Whenever the patient produced the expected SCP shift, he also received a reward in a form of an attractive moving visual-auditory stimulus: a rotating sun animation, green light turning on and applause (Fig. 1a, left top corner). In the first session of block 1 the SCP amplitude threshold, which the patient had to exceed to be rewarded, was fixed to ensure the level of 30 % successful trials in each run. The value was set separately for positive and negative trials. As it was indicated by Kleinnijenhuis (2007), this success level is sufficient to achieve optimal SCP self-regulation learning outcomes. In subsequent sessions the thresholds were continuously updated, using mean SCP values obtained in all positive or negative SCP trials completed on the previous day, so that probability of success remained at approximately 30 %. This approach yielded a stable difficulty levels throughout the whole training.

In the first training block (block 1) the ratio of positive and negative SCP trials in the run was 1:1. In two remaining blocks (blocks 2 and 3), 66 % were positive SCP trials and 33 % were negative SCP trials (ratio 2:1). The 2:1 ratio was set to accelerate the process of elevation of cortical excitation thresholds and, thereby, enhance cortical inhibitory mechanisms (Elbert 1986; Kotchoubey 2000; Lutzenberger et al. 1979; Rockstroh and Elbert 1990; Strehl et al. 2006a). As already stated, cortical excitation thresholds have been found decreased in tinnitus patients (Kropp et al. 2012; Shiraishi et al. 1991).

The positive SCP and the negative SCP trials always came in a fixed order. In the training block 1 a positive SCP trial was always followed by a negative SCP trial. In blocks 2 and 3 after two positive SCP trials, a negative SCP trial was presented. The fixed-order training schedule was applied to facilitate differentiation between the two types of trials. As indicated by Strehl (2009), maintaining one specific brain state for a long duration is a considerable challenge. The fixed order of trials, furthermore, enhances the flexibility of self-regulation mechanisms of cortical excitability, which is desired in patients with tinnitus. According to the authors’ clinical experience, a predictable and an explicit training structure, as opposed to one randomly distributed, accelerates the process of acquisition of SCP self-regulation. The learning process advances more quickly when basic capacities are acquired first, followed by those of higher complexity (Gagne 1970; Sweller 1976). Moreover, the predictable arrangement of the training could help reduce frustration related to uncertainty. This is particularly important in patients with tinnitus who often demonstrate high levels of anxiety and emotional reactivity (Landgrebe and Langguth 2011a; Crönlein et al. 2011; Landgrebe and Langguth 2011b; Greimel and Kröner-Herwig 2011).

#### Transfer Runs

During transfer runs no feedback information was provided. The sun animation and green diode were continuously presented on the Visual Display (Fig. 1b). Whenever the mean SCP amplitude exceeded the threshold fixed at the level that predicted 30 % successful trials (see above), the sun animation rotated, the diode turned on and the applause sound was produced. In each training block (1–3), transfer trials constituted the third run and were arranged in a fixed order. In block 1 the ratio of positive SCP trials to negative SCP trials was 1:1, whereas in blocks 2 and 3 the corresponding ratio became 2:1.

Transfer trials have been implemented in the training protocol, as they have been suggested to develop self-awareness of one’s ability of SCP self-regulation (Kotchoubey et al. 2002). They have also been hypothesized to be more ecologically valid, as compared to regular feedback trials (Zuberer et al. 2015).

#### Transfer of Self-Regulation Training Into Everyday Life

Z.C. also practiced self-regulation skills outside the IPPH. He was asked to sit in front of a PC and use a printed Visual Display of the training procedure, imaging an actual training session at the Institute. It has been suggested that despite no immediate feedback, home practice not only improves the skills trained in the clinical setting but also facilitate the transfer of self-regulation capacities to everyday life (Strehl 2009).

#### EEG Signal Recording

The EEG signal used for Neurofeedback was registered from the active electrode placed at the vertex (Cz position) (Jasper 1958). Reference electrodes were located on linked mastoids and the ground electrode was placed on the back of the neck (C7 position). During the training, electro-oculogram (EOG) was recorded from two additional electrodes attached above and below the right eye The EOG signal was used by the instructor and by the patient himself to monitor and reduce the effect of eye movements on the amplitude of the produced SCPs. The patient was also asked to refrain from eye movements during positive and negative SCP trials and try to blink only during 2-min breaks between them. If eye movements during an SCP trial were too intensive, a red alarm control light turned on in the top left corner of the patient’s training screen suggesting him to stop blinking (Fig. 1a, b). This trial was considered failed due to the eye movement-related artefacts, even if the required SCP amplitude threshold was obtained, and the patient did not receive the reward (the audio-visual stimulus was not presented). The EEG signal recorded in each training session was carefully inspected offline and grand average SCP was calculated based only on artefact-free trials. All these procedures were used to reduce the effect of ocular artefacts on the EEG signal registered during SCP Neurofeedback training sessions.

### Evaluation of the Outcomes of SCP Neurofedback Training

During SCP Neurofeedback training Z.C. was asked not to treat tinnitus by using any alternative methods, and he did not significantly change his daily routine. The outcomes of SCP Neurofeedback training were evaluated with the use of: (a) a detailed clinical interview, (b) the Tinnitus Functional Index (Meikle et al. 2012), (c) measurement of tinnitus characteristics (type, pitch and loudness) with an in-house software programme *Tinnitus,* and (d) QEEG. All these tests were performed before and immediately after SCP Neurofeedback training.

#### Tinnitus Functional Index (TFI)

An experimental Polish version of the Tinnitus Functional Index was completed by the patient to monitor how his subjective perception of tinnitus changed after the training (Meikle et al. 2012). TFI is a self-report instrument measuring the severity of tinnitus and responsiveness to treatment. TFI is divided into 8 subscales, corresponding to 8 main domains of daily life that might be affected by tinnitus: INTRUSIVE, SENSE OF CONTROL, COGNITIVE, SLEEP, AUDITORY, RELAXATION, QUALITY OF LIFE, EMOTIONAL. Responses are given on a 1–10 point scale, reflecting the subjectively perceived effect of tinnitus within the previous week. Scores are calculated separately for each subscale and for the whole questionnaire (an overall TFI). The score is divided by the number of valid questions and then multiplied by 10. This calculation produces a score in the range of 0–100 points and provides a straightforward and an accurate measure. The suggested severity grading for the overall and subscale mean scores is as follows (Henry et al. 2014): <25 points—relatively mild tinnitus, 25–50 points—significant problems, >50 points—very severe tinnitus requiring an aggressive intervention. Henry et al. (2014) consider a 13 point reduction a ‘meaningful change’. The original TFI has an excellent internal consistency reliability (Cronbach’s alpha = 0.97) and a test–retest reliability of 0.78. The tool has, furthermore, a relatively good convergent validity with the Tinnitus Handicap Inventory (*r* = 0.86) and the Visual Analog Scale (a simple scale to assess tinnitus severity (*r* = 0.75), as well as discriminant validity of *r* = 0.56 with the Beck Depression Inventory-Primary Care (Meikle et al. 2012).

#### Measurement of Tinnitus Parameters with Tinnitus Software

The patient was first asked to describe the sound of tinnitus in his own words (e.g. a tone, hiss, noise, etc.), and to estimate its loudness and laterality. Then the *Tinnitus* software was used. The programme, implemented in MATLAB 7.9., was developed to determine the dominant acoustic parameters of the perceived tinnitus. During testing, different auditory stimuli (e.g. pure tone, noise, hiss, etc.) of varying intensity and frequency (pure tones) were presented to the patient via headphones (Sennheiser PMX60) (calibrated according to the ISO-8253-1 norm) in a soundproof chamber. Following the patient’s suggestions, the stimulation was delivered to both ears, starting with pure tones of a very high frequency (20 kHz). Then, the selected tone was intensity-modulated to choose the best match (80 dB HL was the starting point). The procedure was continued until the patient defined the pitch-loudness combination that was most alike the experienced tinnitus. No information was provided to the patient about the parameters of tinnitus, in order to exclude any subjective factors that might have affected the experimental procedure.

#### QEEG Data Acquisition and Analysis

The key tool to assess the outcomes of SCP Neurofeedback training was quantitative electroencephalography (QEEG). The EEG signal was registered in two 3-min blocks. The patient was seated in a comfortable reclining armchair and was asked to relax. In the first block the patient kept his eyes open and fixated on a point of his choice in the room. In the second block his eyes were closed. A 19-channel system by Mitsar Co. Ltd. was used to collect the data. The electrodes were distributed on the head of the patient according to the International 10–20 system (Jasper 1958). The signal was referenced to linked mastoids and the ground electrode was placed in the FCz position. Before the examination started, impedance was measured at all electrodes and remained <5 kΩ for the whole study duration. The registered signal was then analysed quantitatively using WinEEG software (Mitsar Co. Ltd.). Digital pre-processing involved high- and low-pass filtering within the range of 0.5–50 Hz. After-recording eye movements and muscle artefacts were removed with the independent component analysis (ICA) method and visual inspection (Congedo et al. 2008; Makeig et al. 1996). Next, the artefact-free EEG signal was divided into thirty 4.096 s segments and the spectral analysis was performed using the fast Fourier transform (FFT) (Walker 1996). Spectra of the signal amplitude were calculated for both study blocks/conditions (*eyes open* and *eyes closed*). Further quantitative analysis involved the following frequency bands: delta (1–4 Hz), theta (4–8 Hz), low alpha (8–10 Hz), high alpha (10–12 Hz), low beta (12–15 Hz), middle beta (15–18 Hz), and high beta (18–25 Hz). The mean absolute amplitudes of the specific bands were then used to determine their distribution on the head.

## Results

### SCP Neurofeedback Training

**Fig. 2:**
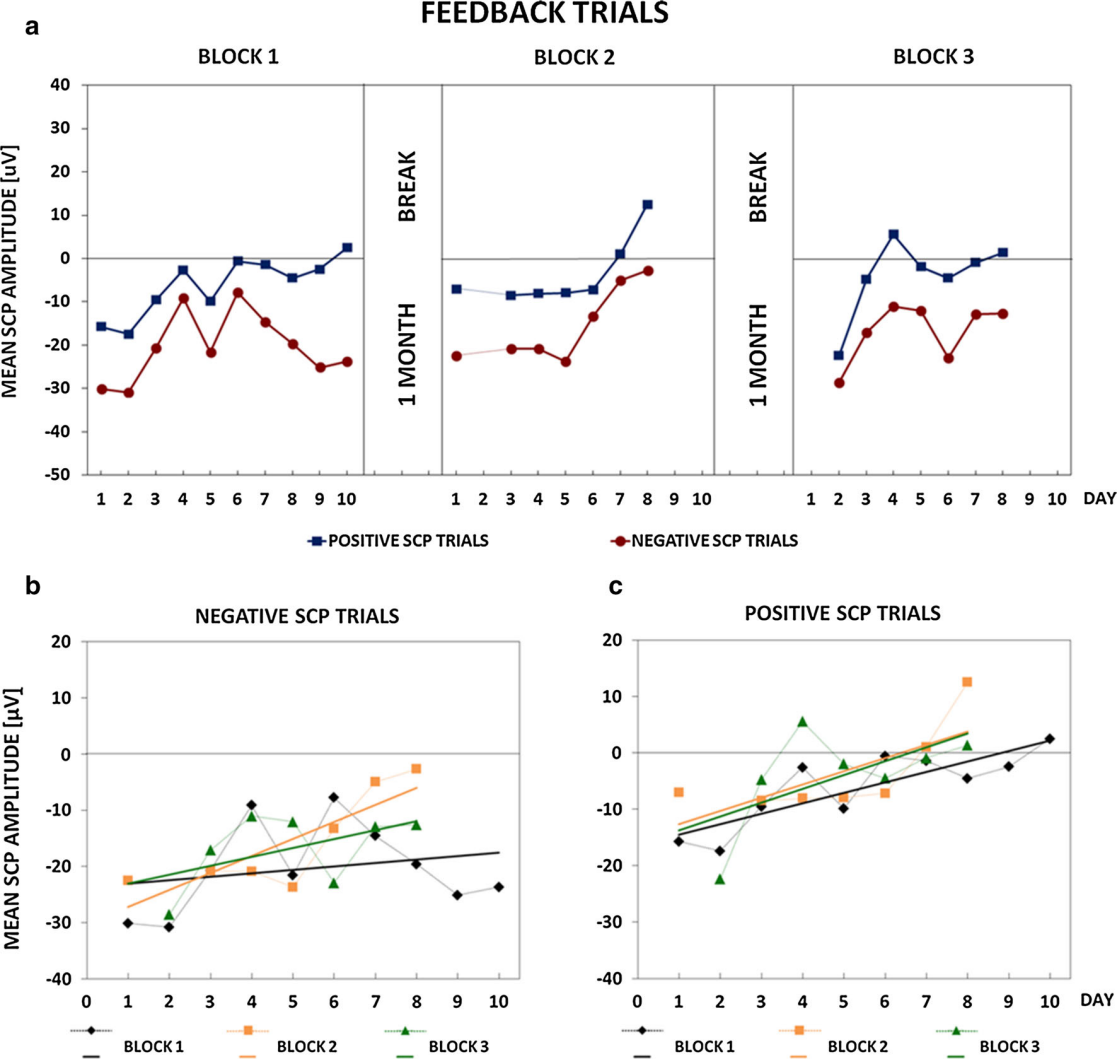
Feedback trials of SCP Neurofeedback training. **a** Mean absolute SCP amplitudes in positive and negative SCP trials in subsequent days of the training. There were 3 training blocks (a maximum of 10 days each) separated by 1-month breaks. On several days the patient was not able to participate or the registered EEG signal was contaminated with artifacts and could not be used for the estimation of mean absolute SCP amplitudes. Therefore, blocks 2 and 3 comprised only 7 days. *Bottom plots* show mean SCP amplitude values obtained in **b** negative and **c** positive SCP feedback trials in all training blocks. *Trend lines* represent the direction of SCP amplitude changes in the course of training days. For several training days mean SCP amplitudes could not be calculated (the rationale see above) and thus some values are missing

Figure 2a depicts learning curves of the patient obtained in the positive and negative SCP feedback trials in subsequent blocks. Linear trends of the mean SCP amplitude in positive and negative SCP trials in each block are also shown (Fig. 2b, c, respectively). Z.C. increased the cortical negativity in negative SCP feedback trials and suppressed negativity in positive SCP trials already in the first 6 days of the training. However, the difference in mean absolute SCP amplitude values of positive and negative trials was unstable (Fig. 2a). From day 7 the difference consistently increased until the end of training block 1 and on day 10 the mean SCP amplitude in positive trials reached a positive value (Table 1). In block 2 the difference remained stable towards day 5, on day 6 and 7 it almost vanished, and again increased at the end of training block 2 (day 8). Positive SCP amplitudes were observed for positive trials in last 2 days of block 2 (day 7 and 8) (Table 1). From day 3 of training block 3 the difference between mean SCP amplitudes in negative and positive trials was stable until the end of the block, with positive trials reaching positive mean SCP values on days 4 and 8 (Table 1). For negative SCP trials there were pronounced positive trends in training blocks 2 and 3, as compared to block 1 (Fig. 2b). As can be seen in Fig. 2c, in positive SCP trials the trend lines towards positivity were in all training blocks with the most substantial, upward trends in block 2 and 3.

**Table 1:** Mean SCP amplitudes in positive and negative feedback trials obtained in subsequent days of SCP Neurofeedback training. Differences between mean amplitudes in positive and negative SCP feedback trials are also presented

Differences between mean SCP amplitudes in positive and negative feedback trials																				
	Block 1										Block 2									
Day	1	2	3	4	5	6	7	8	9	10	1	2	3	4	5	6	7	8	9	10
Mean SCP amplitude in positive feedback trials	−15.7	−17.4	−9.6	−2.6	−9.9	−0.6	−1.4	−4.6	−2.5	2.5	−7.1	–	−8.5	−8.1	−7.9	−7.2	1.0	12.5	–	–
Mean SCP amplitude in negative feedback trials	−30.1	−30.8	−20.7	−9.1	−21.6	−7.8	−14.6	−19.6	−25.1	−23.7	−22.5	–	−20.8	−20.9	−23.7	−13.3	−5.0	−2.7	–	–
Difference	14.4	13.4	11.1	6.5	11.7	7.2	13.2	15.1	22.7	26.2	15.4	–	12.4	12.8	15.8	6.2	6.0	15.2	–	–

	Block 3																			
Day	1	2	3	4	5	6	7	8	9	10										
Mean SCP amplitude in positive feedback trials	–	−22.4	−4.8	5.6	−2.0	−4.5	−0.9	1.4	–	–										
Mean SCP amplitude in negative feedback trials	–	−28.6	−17.1	−11.0	−12.0	−22.9	−12.9	−12.6	–	–										
Difference	–	6.2	12.3	16.6	10.1	18.4	12.0	14.0	–	–										

**Fig. 3:**
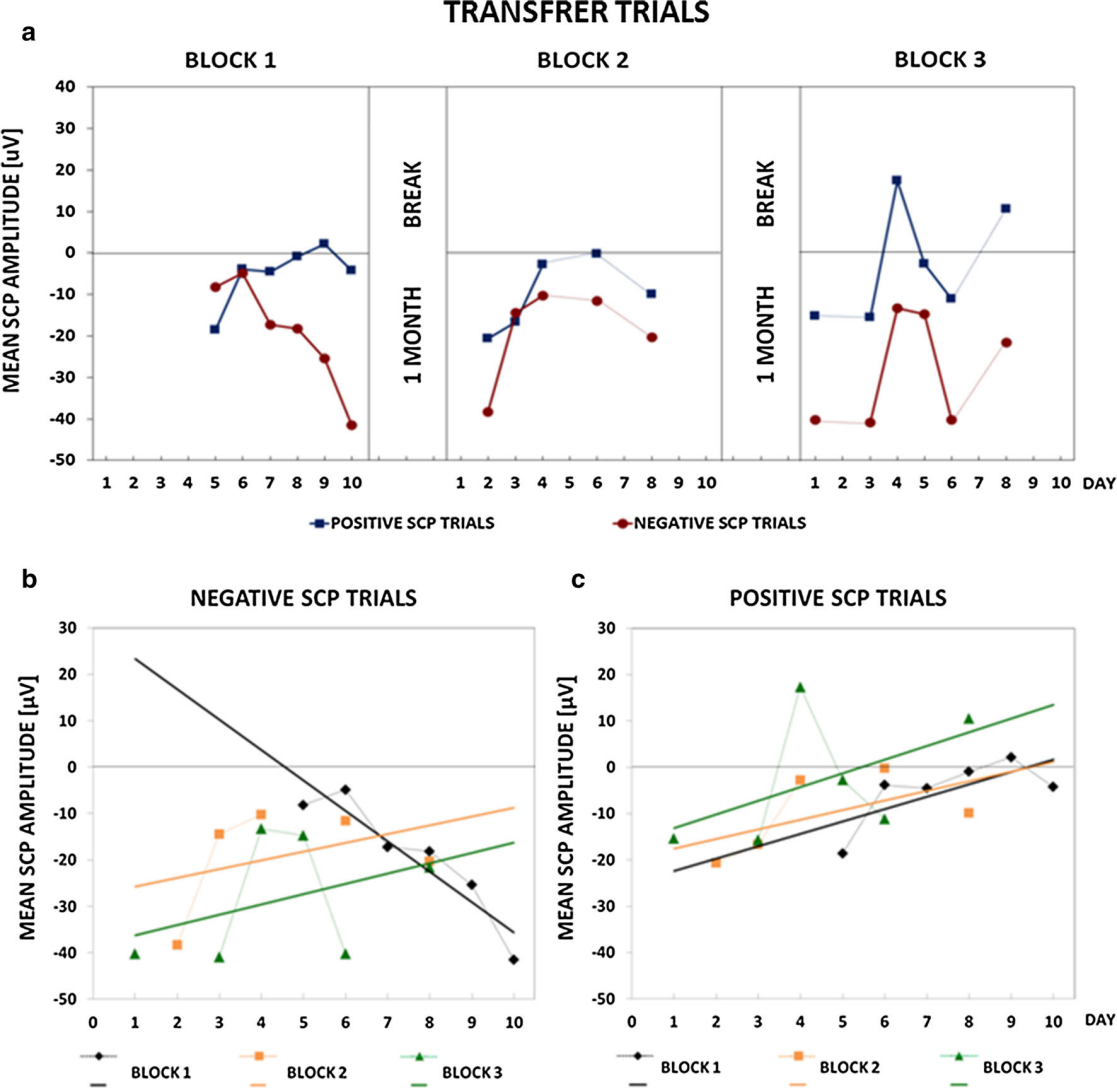
Transfer trials of SCP Neurofeedback training. **a** Mean absolute SCP amplitudes in positive and negative SCP trials in subsequent days of the training. The training comprised 3 blocks separated by 1-month breaks. There were only 6 days of training in block 1 and 5 days in blocks 2 and 3 (of possible 10). On the remaining days, the registered EEG signal was contaminated with artifacts or the patient was unable to perform the training. This data was missing in the estimation of mean absolute SCP amplitudes. *Bottom plots* show mean SCP amplitude values obtained in negative (**b**) and positive (**c**) transfer trials on certain days in all training blocks. The direction of SCP amplitude changes throughout the training is depicted as *trend lines*. Some values are missing for the reason mentioned above

Figure 3 demonstrates how Z.C. learned to self-regulate SCPs in transfer trials (without feedback information). The learning curves in Fig. 3a show that from day 7 until the end of block 1 there was an increasing difference between mean SCP amplitudes obtained in negative and positive trials. The difference decreased but was still apparent in block 2, from day 4 to day 8. In training block 3 the difference between positive and negative trial SCP amplitudes was pronounced and maintained until the end of block 3. Among positive trials the mean SCP amplitude reached positivity on day 9 of block 1 and on day 4 and day 8 of block 3. No positive SCP amplitude values were obtained in block 2 (Table 2). Trends in learning self-regulation of SCPs in individual blocks are shown in Fig. 3b, c. In Fig. 3b, presenting negative SCP trials, there was a clear linear tendency towards negativity in block 1 but in blocks 2 and 3 upward trends were visible. For positive SCP trials (Fig. 3c) linear trends towards positivity, comparable in first two blocks and most pronounced in block 3, were present.

**Table 2:** Mean SCP amplitudes in positive and negative transfer trials obtained in subsequent days of SCP Neurofeedback training. Differences between mean amplitudes in positive and negative SCP transfer trials are also presented

Differences between mean SCP amplitudes in positive and negative transfer trials																				
	Block 1										Block 2									
Day	1	2	3	4	5	6	7	8	9	10	1	2	3	4	5	6	7	8	9	10
Mean SCP amplitude in positive transfer trials	–	–	–	–	−18.6	−3.9	−4.5	−1.0	2.1	−4.3	–	−20.7	−16.7	−2.8	–	−0.2	–	−9.9	–	–
Mean SCP amplitude in negative transfer trials	–	–	–	–	−8.2	−4.9	−17.2	−18.2	−25.4	−41.5	–	−38.3	−14.5	−10.2	–	−11.6	–	−20.3	–	–
Difference	–	–	–	–	10.4	1.0	12.7	17.2	27.5	37.3	**–**	17.6	2.2	7.5	–	11.3	–	10.4	–	–

	Block 3																			
Day	1	2	3	4	5	6	7	8	9	10										
Mean SCP amplitude in positive transfer trials	−15.3	–	−15.6	17.3	−2.7	−11.2	–	10.6	–	–										
Mean SCP amplitude in negative transfer trials	−40.2	–	−40.9	−13.3	−14.7	−40.2	–	−21.6	–	–										
Difference	24.9	–	25.3	30.6	12.0	29.1	–	32.2	–	–										

### Clinical Interview with the Patient

After SCP Neurofeedback training Z.C. reported that tinnitus was still present. He stated, however, that the pitch of tinnitus decreased and the sensation became less intensive. The patient, furthermore, claimed that he learned to pay less attention to tinnitus. In addition, his sleep quality improved and it was easier to fall asleep and get up more rested. The interview revealed that the most important benefit for the patient was that he felt in control of the tinnitus sensation and was able to reduce it by 20–30 %. Furthermore, Z.C. stated he was now able to spontaneously relax and consciously control his muscle tension in any situation. Ultimately, the patient became more relaxed, which in turn improved his contacts with family and friends.

### The Tinnitus Functional Index (TFI)

Before the training the patient’s overall score in TFI was 45 points, whereas after the intervention it decreased to 6 points. This was an 86.6 % improvement which can be considered significant. The results obtained in the 8 subscales of the TFI in the pre- and post-training situations and the percentage change are depicted in Table 3. There was an improvement in all TFI scores.

**Table 3:** Scores obtained in TFI subscales before and after SCP Neurofeedback training. Particular subscales refer to negative effects of tinnitus in the patient’s experience and different domains of daily functioning

The effect of SCP Neurofeedback training on TFI scores				
TFI subscale	Items in subscale	Scores before training	Scores after training	Before–after difference (percentage change)^a^
INTRUSIVE	1, 2, 3	56	26	30 (53.6 %)
SENSE OF CONTROL	4, 5, 6	46	10	36 (78.3 %)
COGNITIVE	7, 8, 9	33	0	33 (97.0 %)
SLEEP	10, 11, 12	63	10	53 (84.1 %)
AUDITORY	13, 14, 15	26	0	26 (96.2 %)
RELAXATION	16, 17, 18	50	0	50 (98.0 %)
QUALITY OF LIFE	19, 20, 21, 22	30	0	30 (96.6 %)
EMOTIONAL	23, 24, 25	60	3	57 (95.0 %)
OVERALL SCORE		45	6	39 (86.6 %)

^a^The before–after difference in TFI was calculated by subtracting the scores after training from those obtained before the training. Percentage of change was determined according to the following formula: 100 % − (scores after training × 100 %)/scores before training

### Measurement of Tinnitus Parameters with *Tinnitus* Software

Before the training the auditory sensation experienced by the patient resembled a pure tone of 12 kHz. The intensity was estimated at 40 dB HL. After the training, the perceived pitch of tinnitus was reduced to 11 kHz and the intensity decreased to 30 dB HL. Both tests were performed using the same procedure and experimental setting.

### QEEG Data

Tables 4 and 5 include detailed comparisons of the QEEG measurements in the *eyes open* and the *eyes closed* blocks before and after SCP Neurofeedback training. The conditions have been presented separately and for subsequent frequency bands.

**Table 4:** Mean absolute amplitudes of specific frequency bands, estimated from QEEG signals registered before and after SCP Neurofeedback training in the *eyes open* condition at electrodes revealing most significant effects. Direction of changes, absolute quantitative difference and percentage signal change have been provided

Frequency band	Brain regions	Electrodes	Before training (μV)	After training (μV)	Direction of change	Absolute difference (μV)/percentage change
Delta (1–4 Hz)	Left fronto-temporal Left temporal Temporo-occipital Left occipital	F7, T3, T5, O1	1.85	1.18	↓	0.67 μV/36.2 %
	Right fronto-temporal	F8, T4	1.14	1.49	↑	0.35 μV/30.7 %
Theta (4–8 Hz)	Left temporal Left temporo-occipital Left occipital	T3, T5, O1	1.15	0.86	↓	0.29 μV/25.2 %
Low alpha (8–10 Hz)	Left temporal	T3	1.54	0.39	↓	1.15 μV/74.7 %
High alpha (10–12 Hz)	Left temporal	T3	1.54	0.9	↓	0.64 μV/41.6 %
Low beta (12–15 Hz)	Left centro-parietal	C3, P3	1.04	0.57	↓	0.47 μV/45.2 %
Middle beta (15–18 Hz)	–	–	–	–	–	–
High beta (18–25 Hz)	Right temporo-occipital	T6	0.7	1.11	↑	0.41 μV/58.5 %
	Left temporal	T3	0.81	1.01	↑	0.2 μV/24.6 %

**Table 5:** Mean absolute amplitudes of specific frequency bands, estimated from QEEG signals registered before and after SCP Neurofeedback training in the *eyes closed* condition at electrodes revealing most significant effects. Direction of changes, absolute quantitative difference and percentage signal change have been provided

Frequency band	Regions	Electrodes	Before training (μV)	After training (μV)	Direction of change	Percentage of change
Delta (1–4 Hz)	Left fronto-temporal Left temporal Left temporo-occipital Left occipital	F7, T3, T5, O1	1.82	1.29	↓	0.53 μV/29.2 %
	Right fronto-temporal	F8	1.60	1.27	↓	0.33 μV/20.7 %
Theta (4–8 Hz)	Left fronto-temporal, Left temporal, Left temporo-occipital	F7, T3, T5	1.47	1.09	↓	0.38 μV/25.9 %
Low alpha (8–10 Hz)	Left fronto-temporal Left temporal Left temporo-occipital Left occipital	F7, T3, T5, O1	3.79	3.02	↓	0.77 μV/20.3 %
	Right occipital	O2	5.45	4.81	↓	0.64 μV/11.8 %
	Right frontal Right fronto-temporal	F4, F8	1.90	1.48	↓	0.42 μV/22.1 %
	Right temporo-occipital	T6	5.45	6.02	↑	0.57 μV/10.5 %
High alpha (10–12 Hz)	Right occipital	O2	2.90	3.47	↑	0.57 μV/19.7 %
	Left temporal	T3	1.75	1.29	↓	0.46 μV/26.3 %
Low beta (12–15 Hz)	Left centro-parietal	C3, P3	1.25	1.03	↓	0.22 μV/17,6 %
Middle beta (15–18 Hz)	–	–	–	–	–	–
High beta (18–25 Hz)	Left temporal	T3	1.15	0.82	↓	0.33 μV/28.7 %

#### Eyes Open

**Fig. 4:**
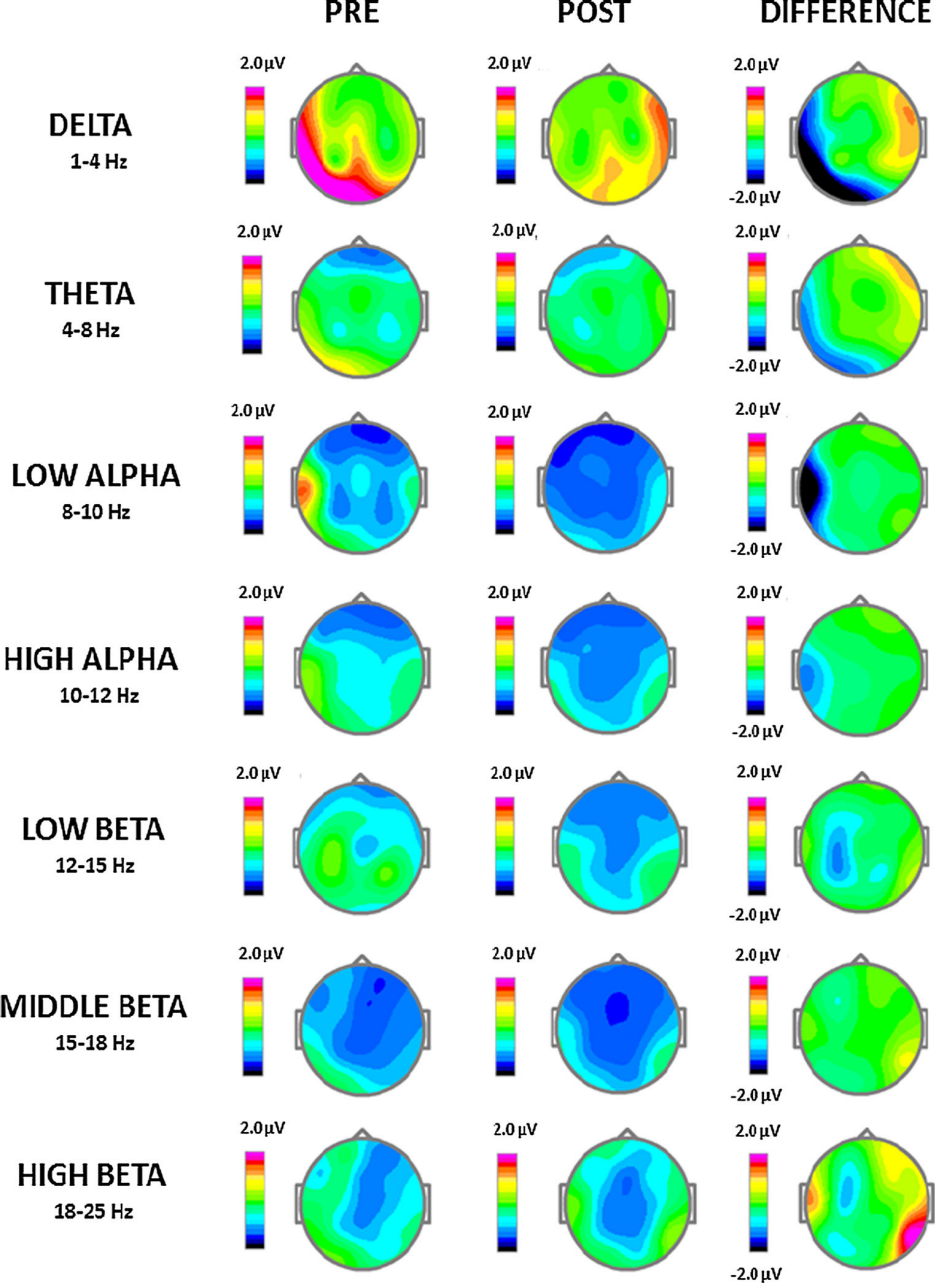
Pre and post-SCP Neurofeedback training two-dimensional (2D) maps of the absolute amplitude distribution for subsequent frequency bands: delta (1–4 Hz), theta (4–8 Hz), low alpha (8–10 Hz), high alpha (10–12 Hz), low beta (12–15 Hz), middle beta (15–18 Hz) and high beta (18–25 Hz) computed from the EEG signal recorded in the *eyes*-*open* condition. The differential amplitude spectra for each frequency band are also provided. The maps were calculated by subtracting absolute amplitude spectra of the signal recorded after SCP Neurofeedback training from the spectra of the signal acquired in the same condition before the training (Color figure online)

**Fig. 5:**
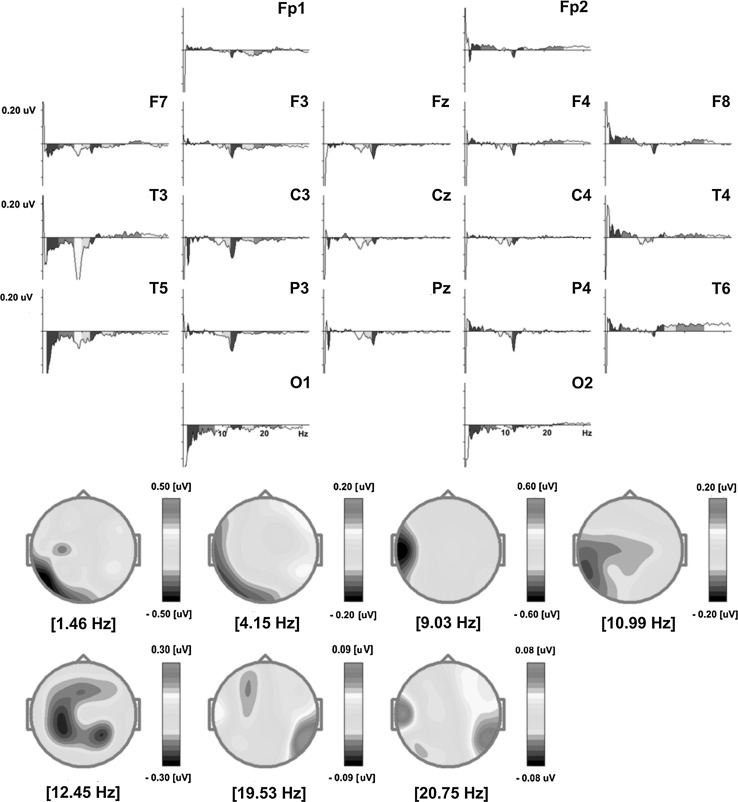
The *upper* part of the figure shows differential absolute amplitude spectra of the EEG signal recorded in the *eyes*-*open* condition calculated separately for each electrode. Particular spectra were computed by subtracting the amplitude spectra of the signal recorded after the SCP Neurofeedback therapy from the amplitude spectra of the signal recorded before the therapy. The *lower* part of the figure presents maps of distribution of frequencies which were most significantly altered after the therapy (Color figure online)

After training, the QEEG analysis demonstrated reduced mean absolute amplitude of the delta frequency band (1–4 Hz) in the left fronto-temporal, temporal, temporo-occipital and occipital brain regions (Fig. 4). The effect was visible at electrodes F7, T3, T5 and O1 and most apparent for 1.46 Hz in the left temporo-occipital and occipital areas (Fig. 5, Table 4). At the same time, a minor increase of the delta band mean absolute amplitude after training was observed at electrodes F8 and T4 (in the fronto-temporal area of the right hemisphere) (Fig. 4; Table 4). The mean absolute amplitude of the theta frequency band (4–8 Hz) dropped after training, predominantly at electrodes T3, T5 and O1 in the left temporal, temporo-occipital and occipital brain areas (Figs. 4, 5; Table 4). The most evident difference was observed for 4.15 Hz (Fig. 5). A further decrease in the mean absolute amplitude was seen for both low (8–10 Hz) and high (10–12 Hz) alpha frequency bands, especially in the left temporal area at electrode T3 (Figs. 4, 5; Table 4). The largest decreases were measured for 9.03 Hz and 10.99 Hz (Fig. 5). These were the most significant effects of the training, with a 74.7 % change of the mean absolute amplitude of the EEG signal for the low alpha frequency band (Table 4). After training, there was also a reduced low beta amplitude (12–15 Hz) found, predominantly in the left centro-parietal regions (Figs. 4, 5). The mean absolute amplitude of the signal at electrodes C3 and P3 considerably diminished (Table 4), which was most evident for 12.45 Hz (Fig. 5). At the same time, after training there were relatively significant increases of the high beta mean absolute amplitude (18–25 Hz) present in the right temporo-occipital area at electrode T6, together with a less pronounced rise in the left temporal region at electrode T3 (Fig. 4; Table 4). The major amplitude increase in the high beta frequency band was observed for 19.53 and 20.75 Hz (Fig. 5). There were no training-related effects observed with respect to the middle beta frequency band amplitude (Fig. 4; Table 4).

#### Eyes Closed

**Fig. 6:**
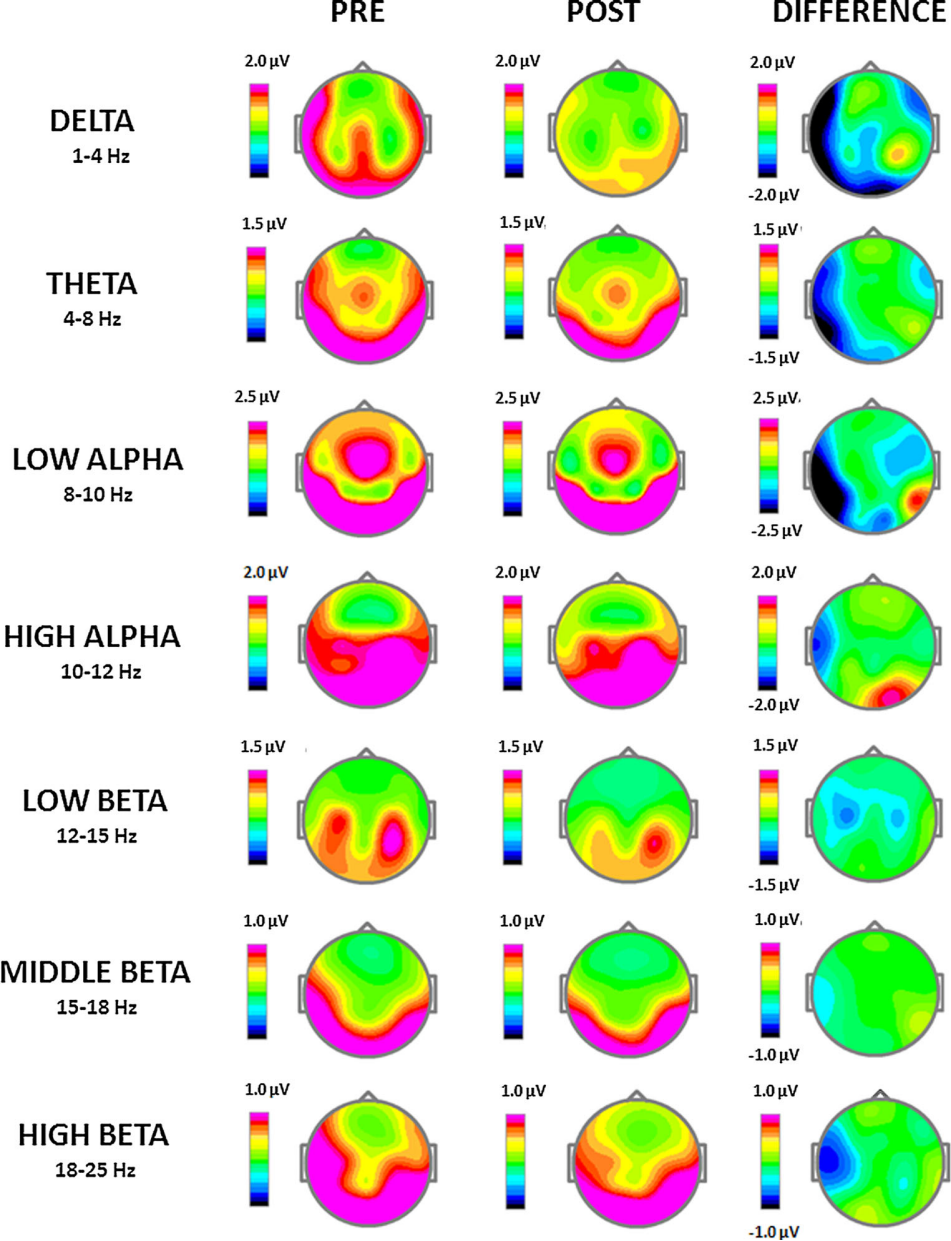
Pre and post-SCP training two-dimensional (2D) maps of the absolute power distribution for subsequent frequency bands: delta (1–4 Hz), theta (4–8 Hz), low alpha (8–10 Hz), high alpha (10–12 Hz), low beta (12–15 Hz), middle beta (15–18 Hz) and high beta (18–25 Hz) were computed from the EEG signal recorded in the *eyes*-*closed* condition. The differential absolute spectra for each frequency band are also provided. The maps were calculated by subtracting absolute amplitude spectra of the signal recorded after the SCP Neurofeedback therapy from the spectra of the signal acquired in the same condition before the therapy (Color figure online)

**Fig. 7:**
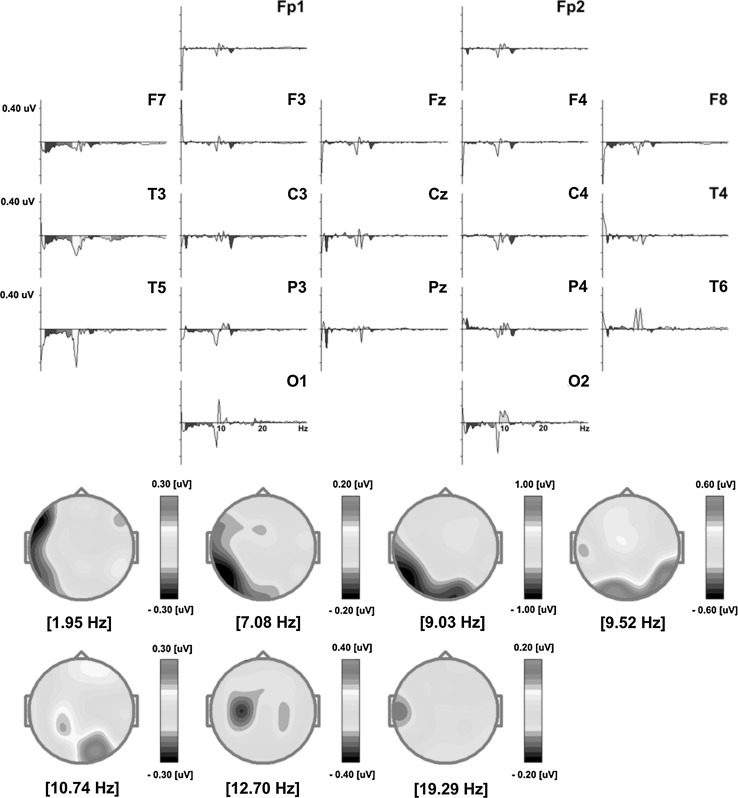
The *upper *part of the figure shows differential absolute amplitude spectra of EEG signals recorded in the *eyes*-*closed* condition calculated separately for each electrode. Particular spectra were computed by subtracting the amplitude spectra of the signal recorded after SCP Neurofeedback training from the amplitude spectra of the signal recorded before the therapy. The *lower *part of the figure presents maps of distribution of frequencies which were most significantly altered after the training (Color figure online)

The QEEG analysis revealed delta band (1–4 Hz) as the one whose amplitude was mostly affected by the SCP Neurofeedback training (29 % change in the amplitude). The effect was most visible at electrodes F7, T3, T5 and O1 in the left fronto-temporal, temporal, temporo-occipital and occipital brain regions (Figs. 6, 7; Table 5). As indicated in Fig. 7, the greatest decrease in the mean absolute amplitude following training was observed for 1.95 Hz in the left fronto-temporal area of the brain. A similar, although less pronounced, reduction of the delta band amplitude was demonstrated in the right frontal areas (Fig. 6; Table 5). The amplitude of the theta frequency band (4–8 Hz) was also significantly decreased after the training. As depicted in Table 5, the effect was most visible at electrodes F7, T3 and T5, i.e. in the left fronto-temporal, temporal and temporo-occipital brain areas (Figs. 6, 7). The largest difference occurred for 7.08 Hz in the left temporo-occipital area (Fig. 7). After SCP Neurofeedback training, the mean absolute amplitude of the low alpha frequency band dropped as well, as measured at the left fronto-temporal, temporal, temporo-occipital and occipital electrodes (F7, T3, T5 and O1), as well as at electrodes O2 and F4, F8 corresponding to the right occipital, frontal and fronto-temporal areas (Figs. 6, 7; Table 5). The effect of the low alpha amplitude reduction was most evident for 9.03 Hz in the left temporo-occipital and occipital areas (Fig. 7). At the same time, focal and limited increases were observed for the low alpha mean absolute amplitude at electrode T6 in the right temporo-occipital region, with the largest increase for 9.52 Hz (Figs. 6, 7; Table 5). There was also a local increase in the high alpha (10–12 Hz) amplitude in the right occipital region (electrode O2) (Fig. 6; Table 5) and the effect was most profound for the 10.74 Hz frequency (Fig. 7). At the same time, in the left temporal region at electrode T3 a considerable drop of the high alpha absolute amplitude was detected (Fig. 6; Table 4). The effect for the low beta (12–15 Hz) was observed predominantly in the left centro-parietal regions (C3 and P3) and was most evident for 12.70 Hz (Figs. 6, 7; Table 5). Importantly, for high beta (18–25 Hz), there was a considerable reduction (by 28.7 %) of the amplitude in the left temporal area (T3) (Fig. 6; Table 5), especially for 19.29 Hz (Fig. 7).

## Discussion

According to our best knowledge, the current work is the first in which SCP Neurofeedback training was used to mitigate tinnitus-related symptoms. We showed that after SCP Neurofeedback training the patient reported suppression of tinnitus sensation and considerable improvement of quality of daily life. These benefits of the therapy were accompanied by changes in resting-state bioelectrical activity (Figs. 4, 5, 6 and 7; Tables 4, 5). During the training Z.C. was able to self-regulate the cortical excitability which could be evidenced by substantial differences in the mean SCP amplitude between the positive and negative trials in both feedback and transfer (no-feedback) conditions (Figs. 2a, 3a). The linear trends in Figs. 2 and 3, showing how the mean SCP amplitude changed across training blocks, indicate an increase of the amplitude in almost all training blocks (except for the first blocks in the negative trials, Figs. 2b, 3b). Such increasing tendency may suggest elevation of the cortical excitation threshold across sessions which is the desirable effect of SCP Neurofeedback training protocol used in this study. Therefore, we may conclude that in a single patient an increased threshold of the cortical excitability co-occurred with specific changes in resting state QEEG patterns and, more importantly, with subjectively reported tinnitus suppression and reduction of tinnitus-related distress.

### Self-Regulation of SCPs

Considering the SCP feedback trials, at the beginning of the training, difference in the mean SCP amplitude between positive and negative trials was present but yet unstable (Fig. 2a). This is not surprising since the patient had just started to learn self-regulation of SCPs. Some authors (e.g. Rockstroh 1987; Rockstroh et al. 1990; Siniatchkin et al. 2000a) suggest that healthy subjects acquire an ability to control SCPs already during the first 2 days of the training. However, no normative data about SCPs and their self-regulation is available Thus, it is hard to determine how long the learning process to differentiate between two brain states is and how this process is dependent on a subject, his own motivation and ability to self-control (Siniatchkin et al. 2000a, b).

A regular pattern of the SCP control was first visible towards the end of training block 1 and throughout most of training block 2 (with the exception of last days in the block). This may indicate that Z.C. was able to self-regulate of SCPs, i.e. to differentiate between the positive and negative SCP trials. However, since at the end of the second training block (Fig. 2a) the mean SCP amplitude increased even in the negative trials. A comparable effect, although in an opposite direction, was observed in ADHD patients participating in SCP Neurofeedback training with a 2:1 ratio between the negative SCP and the positive SCP trials (Strehl et al. 2006b; Strehl 2009). Participants mistakenly produced SCPs of a moderate negative polarity during positive SCP trials, while the performance in the negative SCP trials was correct (i.e. strong negative polarity was generated). At the start of training block 3 SCP mean amplitudes in positive and negative trials were both negative and there was only little visible difference between the SCP amplitudes between the trials (Fig. 2a). However, in the final days of the training the difference in the mean SCP amplitude between the positive and negative trials again increased. The patient seemed to have quickly recalled the skills acquired in the two previous training blocks and his performance remained stable until the end of the training. The complex learning curve converges with other studies reporting highly variable individual learning performance in various training programs of self-regulation of cortical activation, involving not only SCPs (for a review see: Zuberer et al. 2015).

In the current study linear trends in acquiring the ability to self-regulate cortical excitability were also determined to illustrate how the mean SCP amplitude changed during particular blocks and the whole SCP Neurofeedback training (Fig. 2, lower row). In the negative SCP trials in block 2 and 3, there were upward trends only in negative values of the SCP amplitude (Fig. 2b), whereas in the positive trials the trends towards positivity were also determined on the basis of some positive values (Fig. 2c). Such an increase in the mean SCP amplitude may reflect an elevation of the cortical excitation threshold which was the desired effect of the therapy. The patient, however, still achieved mainly negative SCP amplitudes in positive trials of consecutive training sessions (Fig. 2b, c). Some authors (Kotchoubey 2000; Strehl et al. 2006b) suggest that for healthy subjects reducing the cortical negativity is more difficult than increasing negativity. Thus, we may expect that for the patient in our study, who also had negative SCP amplitudes at the start of the therapy (Figs. 2, 3a), negativity suppression would be even more challenging.

Considering negative and positive SCP trials, (Fig. 2, lower row), in the training block 1, the mean SCP amplitude was unstable which is reflected in the trend lines but even better seen when analyzing the mean SCP amplitude values in consecutive days of the training. The more pronounced trends towards positivity appeared only in block 2 and 3 and could be considered as sign of increasing cortical excitation threshold. The differences in the mean SCP amplitude between block 1 and other blocks may, at some extent, be caused by the higher proportion of the positive-to-negative trials (2:1) in last two blocks. Increasing number of positive trials was intentional since we want to facilitate increasing of the cortical excitation threshold hoping that it helps to attenuate the neuronal synchronization underlying tinnitus and alleviate tinnitus-related symptoms. It is worth to mention that similar proportion of the trials was also used by other authors in SCP Neurofeedback trainings in ADHD (Leins et al. 2007; Strehl et al. 2006b). In this case, however, there were more negative than positive trials to favor increasing cortical negativity. Therefore, this is the reversed condition compared to our study aiming at negativity suppression.

Comparing the mean SCP amplitude in negative (Fig. 2b) and positive (Fig. 2c) trials of the last two blocks we cannot claim that predominance of the positive trials largely contributed to an increase of the amplitude. If that was indeed, the upward trends in the positive trials would be more pronounced than those observed in our study. Otherwise, the trends are noticeable, but not as strong as it was expected. Since suppressing negativity is considered as more difficult process than increasing negativity (Kotchoubey 2000; Strehl et al. 2006b), perhaps more trials in SCP Neeurofeedback training are needed to achieve this effect.

Finally, the comparison of changes in the mean SCP amplitude between blocks in both negative and positive trials, may indicate that the learning process was actually involved. Specifically, block 2 always started from the SCP amplitude value similar to this obtained last day of previous block (Fig. 2b, c). Furthermore, for the negative trials (Fig. 2b) in block 2 the mean SCP amplitude began to increase noticeably from the fifth day of the training whereas in block 3 there was a visible SCP amplitude increase already at the beginning of the block and from this moment the amplitude remained stable to the end of the training (Fig. 2b). Comparably in the positive SCP trials (Fig. 2c) there was a considerable increase of the amplitude from the sixth day of block 2 and from the third day of block 3. All these findings may indicate that the patient could maintain an elevated cortical excitation threshold even after a month break.

The achieved SCP self-regulation was more pronounced in the feedback, than in the transfer trials (Fig. 3). This is frequently reported effect in the literature (e.g. Drechsler et al. 2007; Konicar et al. 2015; Leins et al. 2007; Liechti et al. 2012; Siniatchkin et al. 2000a; Strehl et al. 2006b; Strehl 2009). The transfer trials’ outcomes should be, however, interpreted with caution since the learning curves and trends were determined based on the mean SCP amplitudes obtained in only few sessions in each block. Comparable to other studies (e.g. Strehl et al. 2006b, Strehl 2009) we also applied smaller (25 %) number of transfer trials than feedback ones (75 %). As a result, after artefacts removal, less EEG data was analyzed to calculate the mean SCP amplitude on each training day and the outcomes of some sessions were missing (Fig. 3a). Thus, we are far from any unequivocal conclusions about the course of SCP self-regulation learning in transfer trials.

What we can see after inspection of Fig. 3a is a considerable difference between the mean SCP amplitude in the negative and positive trials which was from the seventh day of block 1, from the fourth day of block 2 and during the whole block 3 (Fig. 3a). It was especially evident at the end of the training, when Z.C. was able to differentiate between the positive and negative trials already from the first session of the third block. This is a desirable outcome, especially in case of the SCP transfer trials, since acquiring the ability to self-regulate of SCPs without feedback, may be considered as prerequisite for applying this skill in everyday life (Rockstroh et al. 1993). In training block 1 a clear trend line downwards was observed showing increasing cortical negativity (Fig. 3b). The trends towards positivity were observed in block 2 and 3, and in the latter block, the SCP amplitude was unstable with a large increase on day 4 and equally large decrease on day 6. At the end of the block 3 the SCP amplitude again raised. Similar pattern of changes in the SCP amplitude on consecutive days of block 3 was also found in the positive trials (Fig. 3c). The large discrepancy between SCP amplitude values during this block 3 may reflect unstable cortical excitation threshold in the patient.

We did not observe a considerable effect of increasing proportion of the positive to negative transfer trials in block 2 and 3 (2:1 as compared with block 1 with the 1:1 ratio) on the mean SCP amplitude. We could only say that in case of the negative trials the change of positive-to-negative ratio in last two blocks co-occurred with the upward trends as contrasted with the trend line downwards in the first block (Fig. 3b). However, it is hard to say that it is an effect of the higher positive-to-negative ratio in the last two blocks. Besides, in case of the positive trials, both the trends in block 1 and 2 were comparable and positively directed (Fig. 3b).

### Improvement in Tinnitus Perception and Daily Living

#### Clinical Interview

As stated by the patient during a clinical interview, after the training the severity of tinnitus alleviated, the sensation became more controllable and affected the life of the patient to a lesser extent. The patient reported better cognitive and emotional functioning and an improved social life.

#### Tinnitus Functional Inventory

The results of the TFI confirmed the benefits reported by the patient after the SCP Neurofeedback training. The reduction of the overall TFI score by 86.6 % (from 45 before to 6 points after therapy) (Table 3) indicates a considerably decreased severity of tinnitus (Meikle et al. 2012). According to Meikle et al. (2012), TFI < 25 indicates a relatively mild tinnitus, requiring little or no intervention. However, since an experimental Polish version of TFI was applied, the 6 point score can only be referred to the maximum score of 100 points, which still suggests a minor handicap. As the results in the TFI subscales revealed, the therapy positively influenced many domains of the patient`s daily life. The most apparent improvement was present for the following subscales: RELAXATION, COGNITIVE, QUALITY OF LIFE, AUDITORY, and EMOTIONAL (>90 % change; Table 3). These effects of SCP Neurofeedback training suggest a better ability to relax and have good quality rest. It seems that tinnitus no longer disturbs the patient in a peaceful and quiet surrounding. According to TFI outcomes, Z.C. finds it easier to fall asleep, has better quality of sleep which is deeper and calmer, and feels more rested in the morning (the SLEEP subscale, >84 % change; Table 3). Apparently Z.C.’s views of the future have become more optimistic, as he seems to experience less emotional tension and a significantly improved quality of life. Moreover, it can be assumed from the TFI scores that tinnitus affects the patient’s cognitive functions to a lesser extent and he finds it easier to focus on a task with better cognitive clarity. TFI outcomes, furthermore, suggest that the interference of tinnitus with hearing and understanding speech has considerably diminished. As indicated with a >78 % change in the SENSE OF CONTROL subscale score (Table 3), after the training the patient deals better with the perceived tinnitus and finds it easier to ignore it. However, he is still aware of the sensation which sometimes triggers irritation (INTRUSIVE subscale, >53 % change) (Table 3).

#### Measurement of Tinnitus Parameters with Tinnitus Software

The positive outcomes of SCP Neurofeedback training were also observed in terms of tinnitus characteristics, as assessed by means of the in-house *Tinnitus* software. The decrease of tinnitus loudness by 10 dB was achieved after completing the therapy. This improvement seems significant, especially since the tinnitus parameters were assessed with a relatively objective tool. Moreover, the measurements were performed by a trained operator and the patient was not informed about the parameters of tinnitus determined before and after the therapy. Similar findings were revealed by Davis et al. (2007) who obtained a 12.1 dB improvement in the minimum masking level (the lowest level at which noise masks out the tinnitus sensation) in patients attending Neuromonics Tinnitus Treatment (NTT). This tinnitus therapy method is based on desensitization with the use of auditory stimulation customized for a patient`s hearing and tinnitus profile. A comparable decrease of the tinnitus loudness level was also reported by Lugli et al. (2009) in patients who participated in the windowed sound therapy (WST) during which they were presented with a white noise stimulation band-stopped (notched) around the individual tinnitus central frequency. These authors measured changes in tinnitus loudness using a method similar to that applied in the current study, and found a 12 dB decrease after the therapy.

In the present study not only did the loudness but also the pitch of the patient’s tinnitus decrease following SCP Neurofeedback therapy. This effect might have several causes. It could simply result from discrepancies between the auditory stimuli generated by the *Tinnitus* program and the tinnitus sound perceived by the patient during the evaluation before and after the therapy. This has already been reported as problematic by other authors when assessing the effectiveness of a tinnitus training (Lugli et al. 2009). A true frequency shift of the perceived tinnitus could however, have also occurred, taking into account the high complexity and the sometimes multi-frequency nature of tinnitus (Norena et al. 2002). Although the patient recognized his own tinnitus as a pure tone, it cannot be excluded that, in fact, the perceived sensation consisted of several closely overlapping tones. Therefore, in subsequent tests aimed to identify the tinnitus characteristics (as was the case in the present study) the matching of tinnitus to externally applied sound samples may not have been accurate and also result, to some extent, in the observed discrepancies.

Yet another explanation of the observed effect might involve genuine neuroplastic changes in the auditory cortex. These have been demonstrated in patients with tinnitus in a number of studies employing various techniques, both in terms of structural as well as functional reorganization of the brain (De Ridder et al. 2011; Mühlnickel et al. 1998; Norena et al. 2002; Weisz et al. 2007a, b). The goal of many tinnitus therapies is, thus, to reverse these changes and re-establish normal brain activity (Dohrmann et al. 2007; Tass et al. 2012). In the present paper the subjective shift of the tinnitus pitch reported by the patient after SCP Neurofeedback training could have been caused by a spatial shift of increased neuronal synchronisation from the region of the auditory cortex, including neurons coding the tinnitus pitch to another column(-s) with neurons specialized in processing different sound frequencies (Adamchic et al. 2012). A similar effect was also reported by Adamchic et al. (2012) after Acoustic Coordinated Reset Neuromodulations training was applied, who observed changes in the tinnitus pitch in patients, along with a decrease in its loudness and intrusiveness. The authors suggested that before the therapy the tinnitus-related neuronal synchronization in the auditory cortex was strongly associated with engagement of brain structures subserving emotional processing (cf. the limbic system). A dedicated therapy might have, therefore, induced cortical functional reorganization and thereby decreased the activation of the limbic system. After therapy, neuronal connections between emotionally-related structures and the region of the auditory cortex where the ‘new tinnitus’ is generated are only little developed and/or are relatively weak. This, in turn, renders the ‘new tinnitus’ sensation less aversive. Consequently, the newly established post-training activity in the auditory cortex also has a greater chance to be more rapidly attenuated, as the ‘new tinnitus’ is no longer consciously perceived and finally completely habituated (Adamchic et al. 2012). We assume that a similar mechanism could have occurred in the patient participating in the current study following SCP Neurofeedback training.

### Changes of Resting State Bioelectrical Brain Activity-QEEG

Numerous previous studies have reported abnormal resting state bioelectrical brain activity patterns in patients with tinnitus (Moazami-Goudarzi et al. 2010; Weisz et al. 2007a, b; Milner et al. 2014). Moreover, many authors showed that various therapeutic approaches might make the spontaneous brain activity ‘closer to normal’ (Adamchic et al. 2012; De Ridder 2007; Dohrmann et al. 2007). In the present study there were many positive changes of the bioelectrical brain activity revealed after SCP Neurofeedback training.

A comparative QEEG analysis of signals recorded before and after treatment showed that delta and theta band mean absolute amplitudes were reduced both in signals recorded in the *eyes open* and the *eyes closed* conditions (Figs. 4, 5, 6 and 7; Tables 4, 5). An abnormally increased delta frequency band amplitude has been shown in tinnitus patients and believed to result from missing inputs from the lower auditory structures, including the inner ear (Moazami-Goudarzi et al. 2010; Weisz et al. 2005; Weisz et al. 2007b). As the animal experiments also showed, the amplitude of the delta band increases in the cortical areas deprived of afferent signals (Steriade 2006). In humans this is often the case after stroke when a certain brain region is isolated from others and from the incoming sensory information (Sainio et al. 1983). A similar effect can be also observed during sleep when the power of the delta waves increases due to a limited inflow of the external stimuli to the cortex (Garcia-Rill 2010).

A limited inflow of information from the periphery might also result in an increased amplitude of the theta band. This effect was found in some QEEG studies in patients with tinnitus (De Ridder et al. 2011; Vanneste et al. 2011).

Moreover, De Ridder (2011) reported that the amplitudes of theta and gamma frequency bands are often positively correlated. The authors explained that in healthy individuals this effect might result from the processing of acoustic information which triggers periodic changes of the operating mode in the auditory cortex. A presentation of a stimulus switches the cortex from resting state (characterized by increased regional alpha amplitude) to the mode of the stimulus analysis characterized by a greater percentage share of the high-frequency gamma band. However, if a certain cortical region is deprived of sensory information, as is the case of tinnitus patients, oscillatory brain activity recorded from this area slows down from alpha to theta range. Furthermore, it has been shown that neurons in regions adjacent to cut-off areas increase their synchronization because of no lateral inhibition from regions without sensory inputs. Many of these neurons are engaged in local processing of acoustic stimuli and communicate by means of short distance connections and thus their stronger synchronization results in an increased power of the high-frequency gamma band (De Ridder 2007, 2011; De Ridder et al. 2011, 2013).

The reported amplitude reduction of the delta and theta frequency bands after SCP Neurofeedback training observed in the current study can be, therefore, interpreted as a positive result reflecting a gradual return to an equilibrium in the auditory cortex, between the local resting-state and task-specific brain bioelectrical activity. Interestingly, the effects found for delta and theta frequency bands in signals recorded in both the *eyes open* and the *eyes closed* conditions after SCP Neurofeedback training were also advantageous in terms of location in the brain space. The most significant amplitude reductions were indicated in fronto-temporal, temporal, temporo-occipital (delta and theta) and occipital (delta) regions in the left hemisphere (Figs. 4, 5, 6 and 7; Tables 4, 5). These areas have been often reported to show abnormal bioelectrical function in patients with tinnitus (De Ridder et al. 2011; Moazami-Goudarzi et al. 2010; Van Der Loo et al. 2009; Viirre 2009; Weisz et al. 2005). Some authors also suggested them as crucial in tinnitus generation. The temporal cortical regions are also believed to be strongly associated with the perceptual aspects of tinnitus (i.e. the type of the sound [noise, squeak] and loudness) (Weisz et al. 2005). At the same time, the frontal and the fronto-temporal areas of the brain have been found to integrate sensory and emotional aspects of the perceived tinnitus, determining its significance for the patient, and playing an important role in directing attention to tinnitus (Jastreboff 1990). The normalization of activity in these cerebral regions observed after SCP Neurofeedback training might, therefore, be related to the observed improvement of the clinical state of the patient and his quality of life.

Besides the aforementioned beneficial effects as regards the amplitudes of theta and delta frequency bands, the low and high alpha band amplitude was also reduced following SCP Neurofeedback training, in the *eyes open* condition, most significantly in the left-hemispheric temporal regions (Figs. 4, 5; Table 4). In signals acquired in the *eyes closed* condition a more widely distributed and a less specific effect was observed, with the low alpha band amplitude decrease in the left fronto-temporal, temporal, temporo-occipital and occipital areas (Figs. 6, 7; Table 5) (an effect similar to changes observed for delta and theta frequency bands). The low alpha amplitude was also reduced in the right frontal and fronto-temporal regions (Fig. 6; Table 5). After the therapy in the *eyes closed* condition we furthermore observed an increased amplitude of low and high alpha frequency bands in the right temporo-occipital area and in the right occipital cortex, respectively (Figs. 6, 7; Table 5). The dedicated SCP training also resulted in modified amplitudes of high-frequency beta oscillations. For the *eyes open* condition, the amplitude of the high beta band increased in left-hemispheric temporal and right-hemispheric temporo-occipital regions (Figs. 4, 5; Table 4). For the *eyes closed* condition the low beta amplitude, however, decreased in the centro-parietal areas and the high beta amplitude was considerably reduced in the left temporal region (Figs. 6, 7; Table 5).

Decreased alpha amplitudes have been previously found in tinnitus patients (Weisz et al. 2005; Weisz et al. 2007b). It has been indicated that the tau rhythm, i.e. brain oscillations within the alpha frequency range produced in the perisylvian regions, was most significantly reduced in tinnitus patients (Lehtelä et al. 1997). In addition, this effect was often accompanied by an increased high-frequency activity in beta and/or gamma frequency ranges (Lorenz et al. 2010). Tau is believed to correspond to local inhibitory processes in the auditory cortex and is associated with activity of inhibitory interneurons which intensifies when the brain shifts from a task-related activity to resting-state (Jensen and Mazaheri 2010). In tinnitus, the reduction of the tau rhythm can, then, indicate faulty inhibition mechanisms. Following auditory deprivation the interneurons in the columns of auditory cortex with reduced inputs are not sufficiently stimulated.

As described above, the increased amplitude of high beta and gamma bands might result from neuronal activity in brain regions adjacent to those deprived of auditory inputs. These areas receive the signal from the inner ear but increase firing and synchronization because of missing lateral inhibition. This abnormal neural activity is perceived as tinnitus and reflected in high beta and gamma frequency bands. These high–frequency rhythms are often recognized as the electrophysiological markers of tinnitus (Weisz et al. 2007a, b).

The existing literature and the very positive feedback provided by the patient participating in the current trial suggest that the effects of SCP Neurofeedback training (especially the increased alpha and the decreased beta amplitudes) are very promising. This is especially true for the increased alpha band amplitude in the temporo-occipital areas that can be indicative of re-establishing the proper inhibition mechanisms in the auditory brain regions. Also the reduced amplitude of the beta frequency band, observed in the *eyes closed* condition, can be associated with the improvement of the patient’s mental condition and the subjective reduction of the perceived tinnitus loudness. Comparable findings were demonstrated by Adamchic et al. (2012), who showed a post-training shift of the tinnitus frequency that correlated with a reduced amplitude of the gamma band.

At the same time, the interpretation of the decreased amplitude of the alpha band in temporal and temporo-occipital areas and the increased amplitude of beta in the left temporal and the right temporo-occipital regions remains challenging (Figs. 4, 5, 6 and 7; Tables 4, 5). Some sleep studies showing significant correlations between reduction of the delta power and an increased power of several other frequency bands (alpha, sigma and beta) can provide a plausible explanation (Benoit et al. 2000). An alternative and a more precise account of the current findings concerns a direct relationship between the alpha band amplitude and the tinnitus sensation. As an example, Vanneste et al. (2010) reported an increased power of low and high alpha in patients with tinnitus. Furthermore, studies by Sedley et al. (2015) revealed a positive correlation between alpha power and tinnitus severity. Specifically, the tinnitus suppression was accompanied with a reduced power of alpha band in brain regions also revealing decreased delta/theta amplitudes. Similar effect has been observed in the current study, which can be considered a positive outcome of SCP Neurofeedback training.

The locally increased amplitude of the beta band might have been caused by an extensive muscle tension, which has been most visible in the temporal and frontal brain regions adjacent to the moving jaw and the forehead (Van de Velde et al. 1998). The increased beta amplitude in temporal brain regions, only observed in the *eyes open* condition, could confirm this interpretation. In this condition the patient is in a general state of wakefulness and is ready to receive stimulation which is accompanied by an increased muscle tension. On the other hand, an effect of increased beta amplitude after training can be also related to the reported reduction of tinnitus. In a recent study by Sedley et al. (2015) significant increases of both gamma (>28 Hz) and beta (20–28 Hz) power can be directly associated with tinnitus suppression. Since studies examining the direct relationship between alpha and beta amplitudes and the tinnitus sensation are scarce, the effect of decreased alpha and increased beta amplitudes after Neurofeedback training seen in the present study require further investigation.

Interestingly, there was a laterality effect observed in the differential absolute amplitude spectra calculated before and after SCP training (Figs. 4, 6, last column). Specifically, the effects for delta, theta, alpha and high beta bands were more pronounced in the left hemisphere. This might have been caused by the left hemispheric dominance observed in the patient before SCP Neurofeedback training both in the *eyes open* and *eyes closed* conditions (Figs. 4, 6, first column).

Similar results were obtained by other authors, e.g. Moazami-Goudarzi et al. (2010), who found cortical generators of delta, theta, alpha and beta bands in tinnitus patients in several auditory and non-auditory regions of the left hemisphere. The results were accounted for by cognitive and emotional factors associated with the presence of tinnitus. This explanation might be also true for patient Z.C. who perceived the tinnitus sensation as intrusive and causing negative emotions. It can only be speculated that the observed laterality effect is associated with the frustration or non-acceptance of the auditory phantom sensation, which is conceptual in nature and, therefore, left-hemisphere dominant.

It is also possible that the left hemisphere advantage is related to an increased control of tinnitus, as it was suggested by Vanneste and De Ridder (2012). They found that modulation of the left ventrolateral prefrontal cortex (VLPFC) by transcranial magnetic stimulation TMS) can modify tinnitus sensation. Another argument supporting the left-hemispheric dominance in tinnitus control comes from a recent study by De Ridder et al. (2015). The authors found that abnormal functional connectivity in the theta band between the auditory cortex and parahippocampal brain regions subserves the subjectively perceived tinnitus loudness and is lateralized to the left side of the brain. The loudness of a phantom sensation might be, however, associated with the amount of distress related to the sound. Thus, the higher amplitudes of both low (delta, theta and alpha) and high frequency (beta) oscillations observed before SCP Neurofeedback training, can be related to tinnitus severity and an increased control of the phantom tinnitus sensation. This control can be exerted with considerably less effort after an effective therapy which contributes to a decrease of the laterality effect. This has been observed in the current experiment (Figs. 4, 6, middle column).

## Conclusions

After SCP Neurofeedback training the patient reported suppression of tinnitus sensation and substantial improvement in various aspects of everyday life. This improvement co-occurred with changes of the resting state bioelectrical activity. SCP Neurofeedback training might be considered as an appropriate method for tinnitus therapy but further group investigations are required.
